# Benefits of preparing for childbirth with mindfulness training: a randomized controlled trial with active comparison

**DOI:** 10.1186/s12884-017-1319-3

**Published:** 2017-05-12

**Authors:** Larissa G. Duncan, Michael A. Cohn, Maria T. Chao, Joseph G. Cook, Jane Riccobono, Nancy Bardacke

**Affiliations:** 1 School of Human Ecology, University of Wisconsin-Madison, Madison, Wisconsin USA; 2 Department of Family Medicine and Community Health, University of Wisconsin-Madison, Madison, Wisconsin USA; 3 Osher Center for Integrative Medicine, University of California, San Francisco (UCSF), San Francisco, California USA; 4 Department of Medicine, UCSF, San Francisco, California USA; 5 Student Nurse Midwifery Program, UCSF, San Francisco, California USA; 6 Mindful Birthing and Parenting Foundation, Oakland, California USA; 7 Department of Family Healthcare Nursing, UCSF, San Francisco, California USA

**Keywords:** Mindfulness, Childbirth, Labor, Fear, Pain, Postpartum depression

## Abstract

**Background:**

Childbirth fear is linked with lower labor pain tolerance and worse postpartum adjustment. Empirically validated childbirth preparation options are lacking for pregnant women facing this problem. Mindfulness approaches, now widely disseminated, can alleviate symptoms of both chronic and acute pain and improve psychological adjustment, suggesting potential benefit when applied to childbirth education.

**Methods:**

This study﻿, the Prenatal Education About Reducing Labor Stress (PEARLS) study, is a randomized controlled trial (RCT; *n* = 30) of a short, time-intensive, 2.5-day mindfulness-based childbirth preparation course offered as a weekend workshop﻿, the *Mind in Labor (MIL)*: *Working with Pain in Childbirth*, based on *Mindfulness-Based Childbirth and Parenting* (MBCP) education.﻿ First-time mothers in the late 3rd trimester of pregnancy were randomized to attend either the MIL course or a standard childbirth preparation course with no mind-body focus. Participants completed self-report assessments pre-intervention, post-intervention, and post-birth, and medical record data were collected.

**Results:**

In a demographically diverse sample, this small RCT demonstrated mindfulness-based childbirth education improved women’s childbirth-related appraisals and psychological functioning in comparison to standard childbirth education. MIL program participants showed greater childbirth self-efficacy and mindful body awareness (but no changes in dispositional mindfulness), lower post-course depression symptoms that were maintained through postpartum follow-up, and a trend toward a lower rate of opioid analgesia use in labor. They did not, however, retrospectively report lower perceived labor pain or use epidural less frequently than controls.

**Conclusions:**

This study suggests mindfulness training carefully tailored to address fear and pain of childbirth may lead to important maternal mental health benefits, including improvements in childbirth-related appraisals ﻿and﻿ the prevention of postpartum depression symptoms. There is also some indication that MIL participants may use mindfulness coping in lieu of systemic opioid pain medication. A large-scale RCT that captures real-time pain perceptions during labor and length of labor is warranted to provide a more definitive test of these effects.

**Trial registration:**

The ClinicalTrials.gov identifier for the PEARLS  study is: NCT02327559. The study was retrospectively registered on June 23, 2014.

## Background

Fear of childbirth poses substantial risks to healthy adjustment from pregnancy through birth and into the early postpartum period. It is related to low childbirth self-efficacy, greater use of pain medication during labor, more unwanted obstetric interventions in labor [1–3], as well as increased risk of postpartum depression (PPD) [4]. Childbirth education courses are the primary mechanism by which pregnant women learn strategies for coping with labor pain, yet childbirth education has limited efficacy for reducing childbirth fear and in some cases may even cause women to doubt their ability to cope with childbirth [5], increasing fear. With over 3.9 million births in the United States (U.S.) per year [6], innovative and accessible interventions for addressing childbirth fear and pain are critically needed. Mindfulness training – long used as a method for promoting coping with chronic pain [7, 8] and shown to be beneficial for acute pain [9, 10] – provides a novel and promising strategy for preparing women for childbirth.

Childbirth fear, including its hallmark indicator of low childbirth self-efficacy, predicts poorer labor and delivery related outcomes. Alehagen and colleagues [1] compared primiparous and multiparous women and found that first time mothers had higher levels of fear of childbirth. Fear of childbirth in early labor predicted the total amount of pain medication used during the labor. Data from the Danish National Birth Cohort of over 25,000 nulliparous women indicate that fear of childbirth during pregnancy, particularly in late pregnancy (around 31 weeks), was related to a higher risk of emergency Cesarean section, controlling for other risk factors (i.e., weight gain, birth weight, head circumference, and duration of pregnancy) [2]. These findings suggest the potential utility of intervening with first time mothers in the 3rd trimester to reduce childbirth fear and pain and improve perinatal outcomes.

Fear of childbirth and negative birth experiences are linked with depression, all of which can lead to poorer mother-infant adjustment in the perinatal and postpartum periods. In a sample of 89 women, pain catastrophizing scores collected during active labor prior to the administration of analgesia predicted both postpartum depression and social functioning 6 weeks post-birth [11]. Antenatal depression often goes undiagnosed and untreated; available treatments for PPD include psychotherapy and anti-depressant medication, but barriers to therapy (e.g., stigma concerns, time/expense involved) and risks of medication to breastfeeding infants restrict access to those treatments [12]. Data from trials of mindfulness interventions have consistently shown a beneficial effect on depression symptoms and other mood disorders [13–15]. Incorporating mindfulness into childbirth education available to the general public offers pregnant women experiencing depression symptoms, or otherwise at-risk for PPD, an alternative, stigma-free strategy for addressing these concerns while protecting their ability to engage in medication-free breastfeeding and sensitive, responsive mother-infant interactions.

### The current study

We developed the *Mind in Labor (MIL): Working with Pain in Childbirth* (author NB), a childbirth education program that teaches mindfulness skills for coping with childbirth pain and fear in a short, time-intensive 2.5-day weekend workshop. We hypothesized that mindfulness training through MIL would: 1) produce an adaptive shift in fear and pain-related appraisals of childbirth, thereby increasing childbirth self-efficacy and reducing pain catastrophizing; 2) lead to lower labor pain ratings, less use of pain medication in labor, and greater birth satisfaction; and 3) lower perinatal depression symptoms and protect against postpartum depression. This study, the Prenatal Education About Reducing Labor Stress (PEARLS) study, is a small, randomized controlled trial (RCT) testing these hypotheses in a comparison of MIL against a treatment-as-usual (TAU) active control condition of standard childbirth education with no mind-body focus.

## Methods

### Participants

**Fig. 1:**
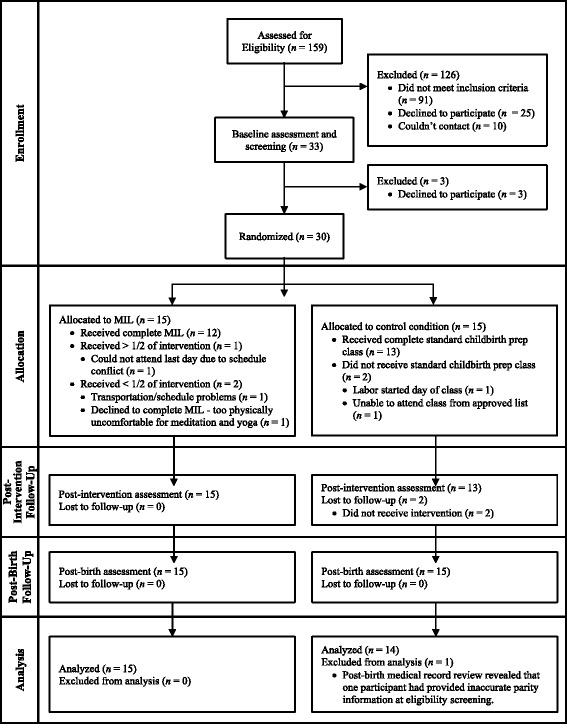
CONSORT flow chart

Inclusion criteria: participants were English-speaking nulliparous women with low-risk, healthy, singleton pregnancies in their third trimester who were planning a hospital birth and willing to be randomized. Exclusion criteria included high-risk pregnancy, extensive prior experience with meditation or yoga practice (brief prenatal yoga did not lead to exclusion), participation in other mind/body childbirth preparation courses (e.g., Hypnobirthing, Bradley Method), or planned Cesarean birth. In terms of race/ethnicity, the eligible, enrolled sample was 18% Latina/Hispanic (*n* = 5, missing = 1); 59% White (*n* = 17); 17% Asian (*n* = 5); 14% Multiracial (*n* = 4); 7% Black/African American (*n* = 2); 3% American Indian/Alaska Native (*n* = 1). More than half of the sample was below area median household income (*n* = 16, 55%); 10% reported household income < $10,000/year (*n* = 3). See Fig. 1 for the CONSORT flow chart of study participation.

### Ethics approval and consent to participate

This study was approved by the University of California, San Francisco (UCSF) Committee for Human Research (Institutional Review Board). All participants gave signed informed consent for participation in research and provided signed Health Insurance Portability and Accountability Act of 1996 (HIPAA) authorization for complete medical record review.

### Procedures

First-time mothers in their third trimester of pregnancy were recruited to the study through provider referrals, as well as fliers and online parenting group recruitment advertisements with language targeting women with typical fears related to childbirth: e.g., “Are you worried about pain during labor?”. Initial eligibility screening was conducted by a brief online survey with follow-up by telephone to capture information related to each of the inclusion/exclusion criteria referenced above. We also collected information regarding intention to use epidural.

To minimize the probability of baseline group differences, randomization was stratified by pre-course intention to use epidural anesthesia and was performed with randomly varying blocks of 2 and 5 using a pre-programmed computer database. A UCSF senior biostatistician not affiliated with the study generated the randomization scheme. The study project manager (JGC) enrolled and consented study participants; group assignment and subsequent debriefing regarding intervention attendance was conducted by him opening a sealed envelope provided by the biostatistician. Data collection was completed online and through medical record review. The remaining study authors (including data analysts) were blinded to participant study condition.

As this was a small study with limited funding, we were constrained to a sample size of *N* = 30 that was selected *a priori* based on sample size recommendations for pilot trials (thus limiting our power to detect effects). Those assigned to the MIL group (*n* = 15) were given a space in a publicly available MIL workshop at UCSF during their third trimester. Tuition for MIL was paid for by the study. Participants assigned to the control condition (*n* = 15) were offered up to $200 USD tuition reimbursement for attending a study-approved, standard hospital- or community-based childbirth education course in the San Francisco Bay Area.

Self-report assessments were conducted at three time points: Time 1 (T1) = 3rd trimester baseline (immediately pre-intervention and pre-randomization) at average gestational age 29.4 weeks (standard deviation; SD = 3.64 weeks); Time 2 (T2) = in the week immediately following the intervention (post-intervention but prior to birth); and Time 3 (T3) at post-birth (postpartum) follow-up (conducted, on average, within six weeks after birth). In addition to providing childbirth education tuition, we offered incentive payments for each assessment (T1 = $40 USD; T2 = $50 USD; T3 = $60 USD), gradually increasing the amount offered to minimize study attrition. Data collection for this study was completed in 2014.

### The mind in labor (MIL): working with pain in childbirth

MIL is a brief intervention for pregnant women and their partners specifically designed to target labor-related fear and pain by teaching tailored mindfulness-based coping strategies. It is a childbirth-specific, short form of the nine-week Mindfulness-Based Childbirth and Parenting program (MBCP) [16] adapted starting in 1998 from Mindfulness-Based Stress Reduction (MBSR) [17]. The MIL course is delivered by professionally certified MBCP instructors and it is held over one weekend (Friday evening and all day Saturday and Sunday) for a total of 18 h of mindfulness training. Mindfulness strategies for coping with labor-related pain and fear are taught through interactive, experiential activities, with periods of didactic instruction.

In addition to standard childbirth preparation topics (i.e. birth physiology), the MIL program includes the following aims and learning objectives: 1) participants are guided to reframe childbirth pain as unpleasant physical sensations that come and go, moment by moment; 2) participants are taught how to uncouple the sensory component of pain from its cognitive and affective components, with the objective of decreasing fear and suffering related to the physical pain of childbirth; 3) participants learn how to be more aware of their own body and fearful reactivity to pain by practicing mindful coping with pain through a pain induction activity with ice; 4) pregnant women and their birth partners develop personalized strategies to best cope interpersonally and provide support to each other throughout the birth process.

To meet these objectives, instruction in formal mindfulness meditation are given during the workshop, including body scan, mindful movement/yoga, sitting and walking meditation, and mindful eating, as well as activities of daily living and pain coping strategies, such as mindfulness of breath, partner touch, body movement, and “sounding” (using low and/or loud vocal tones during periods of intense physical sensation). Additionally there is an inquiry practice between partners exploring fear in general and fear of childbirth in particular and specific mindfulness coping strategies for being with pain with an attitude of acceptance. Participants are provided with handouts and guided audio materials for optional practicing of mindfulness meditation and pain coping strategies at home. In the current study, the course developer (NB), a senior mindfulness teacher and certified nurse midwife, provided facilitation for all MIL intervention participants.

### Active control condition: treatment as usual (TAU) standard childbirth education

Participants assigned to the TAU control condition were provided with a list of study-approved childbirth courses of comparable length and quality to the MIL intervention, but without any mindfulness meditation, mindful movement/yoga, or other core mind/body component (e.g., hypnosis). To form the approved list, we conducted a web search of available San Francisco Bay Area childbirth preparation courses then followed up with direct contact with providers to determine specific content regarding any topics related to mind/body health or stress reduction. If a control group participant could not find a pre-approved course convenient to their location and schedule, we offered to screen their preferred course. In this way, our list of approved courses grew as participants informed us of additional childbirth preparation programs. TAU control participants were strongly encouraged to participate in the control childbirth education with a partner or support person, just as with MIL. In the current study, *n* = 12 of the control participants reported that their partner or spouse attended the course with them.

### Measures

Eligibility screening and all assessments were administered online via the Internet application, SurveyGizmo [18]. The time points when they were administered to collect data for the current study is noted in parentheses.

#### Childbirth self-efficacy (T1, T2)

Childbirth self-efficacy was assessed with the Childbirth Self-Efficacy Inventory (CBSEI; α = .90) [19]. The self-efficacy expectancy items rate how confident respondents feel in their ability to use the behaviors during labor and birth (1 = *Not at all sure* to 10 = *Completely sure*).

#### Maladaptive pain appraisal (T1, T2)

Maladaptive pain appraisal was assessed with the Pain Catastrophizing Scale (PCS; α = .92) [20]. Respondents were asked to reflect on past experiences of physical pain and to rate the degree to which they experience particular thoughts and feelings (e.g., “It’s awful and I feel that it overwhelms me” on a scale of 0 (*Not at all*) to 4 (*All the time*).

#### Perceived pain in labor (T3)

The Visual Analog Scale (VAS) [21] was used to assess perceived labor pain. Participants were asked to retrospectively mark the level of pain they felt for each stage of labor on a 10 cm line representing a continuum of “*no pain*” to “*worst possible pain*.” The VAS is one of the most commonly used pain measures and it has been used successfully to assess labor pain [22]. Participants rated their experiences of pain on the VAS during early labor (until 3–4 cm dilation), during active labor (from 4 cm to pushing), during pushing until birth, and from birth of the baby to delivery of the placenta.

#### Use of pain medication in labor

Use of pain medication in labor was ascertained from medical record review. Epidural/spinal anesthesia and opioid analgesia were coded as dichotomous variables. Use of opioid analgesia was endorsed if any systemic opioid narcotic (e.g., fentanyl, morphine) was administered at any point during labor (before birth), independent of epidural/spinal anesthesia.

#### Birth satisfaction

A modified 24-item version of the Wijma Delivery Expectancy/Experience Questionnaire (W-DEQ; α = .94) [23] was used (T1 - T3) to gauge satisfaction with the birth experience (e.g., : “How happy were you in general during the labor and delivery?”) controlling for W-DEQ expectancies captured prior to labor and delivery. Items were rated on an intensity scale from 1 = *Extremely* to 6 = *Not at all*, and response options are customized for each item (e.g., 1 = *Extremely happy* to 6 = *Not at all happy*). Minor modifications to the scale were made in consultation with obstetric experts on the study team to enhance interpretability and cultural sensitivity of the terminology (e.g., we removed the item asking whether participants imagined they would feel “funny, natural, self-evident, or dangerous” at the time of delivery).

Additionally, we asked respondents to rate their satisfaction with their overall birth experience (T3), as well as with the care they received from healthcare providers during the labor and delivery (T3), on a scale of 1 – 10, with 1 = *Not at all satisfied* and 10 = *Completely satisfied*.

#### Depression

The 20-item Center for Epidemiologic Studies Depression Scale (CES-D; Cronbach’s α ranging from .85 to .90) [24] was used to measure depression symptoms (T1 - T3). The CES-D is widely used and is recognized to be reliable and valid. A score ≥ 16 is the clinical cutoff indicating risk of clinical depression.

#### Mindfulness and mindful body awareness

The Five Facet Mindfulness Questionnaire (FFMQ) [25] was used to assess levels of dispositional mindfulness (a tendency to avoid mindlessness in everyday life) at each time point (T1 - T3). The FFMQ consists of 39 items, yielding subscale scores that measure five elements of mindfulness (observing, describing, acting with awareness, nonjudging of inner experience, and non-reactivity to inner experience; Cronbach’s α ranging from .75 to .91).

The Multidimensional Assessment of Interoceptive Awareness (MAIA) [26] was used (T1, T2) to assess body awareness, which may be an important dimension of mindfulness [27] and particularly relevant for women preparing for childbirth. The MAIA consists of 32 items and measures eight dimensions of interoceptive awareness (noticing, distracting, worrying, attention regulation, emotional awareness, self-regulation, body listening, and trusting; Cronbach’s α ranging from .66 to .87).

## Results

All analyses were conducted as intention to treat analyses with the sample of *n* = 29 (*n* = 14 control and *n* = 15 MIL). Results in Table 1 exclude *n* = 2 participants who did not experience labor for whom the analysis was therefore not valid.

**Table 1:** Crosstabulation of opioid analgesia use by study condition

		Labor pain management: Use of opioid analgesia		Total
		NO	YES	
Study condition	Control	5	***8***	13
	MIL	***9***	4	13
Total		14	12	26

Subsample analyzed: *n* = 26; *n* = 3 participants excluded (*n* = 2 who experienced no labor due to Cesarean births and 1 with missing data)

### Treatment group equivalence

See Table 2 for means and standard deviations for the primary and secondary study outcomes. The two groups (MIL and TAU) were compared at baseline using *t*-tests and found to be equivalent (no statistically significant group differences prior to intervention participation). Of the *n* = 29 study participants, *n* = 4 experienced Cesarean section (*n* = 2 per condition), and among those *n* = 2 experienced no labor (*n* = 1 per condition). Only *n* = 1 participant (in the control condition) had an instrument assisted spontaneous vaginal birth; she used opioid analgesia. The remainder of participants (*n* = 24) had spontaneous vaginal births.

**Table 2:** Descriptive statistics by study condition for primary and secondary outcome variables

	Control			MIL		
	T1: 3rd Trimester Baseline Mean (SD)^a^	T2: Post-Intervention (prior to birth) Mean (SD)	T3: Post-birth Mean (SD)	T1: 3rd Trimester Baseline Mean (SD)^a^	T2: Post-Intervention (prior to birth) Mean (SD)	T3: Post-birth Mean (SD)
CBSEI	197.3 (49.0)	212.0 (35.4)	N/A	165.1 (87.2)	243.3 (41.6)	N/A
PCS	18.7 (8.4)	18.5 (8.6)	N/A	18.5 (10.8)	14.9 (6.4)	N/A
MAIA	2.9 (0.6)	3.0 (0.7)	N/A	2.5 (0.8)	3.1 (0.5)	N/A
FFMQ	3.5 (0.3)	3.6 (0.41)	3.6 (0.55)	3.4 (0.4)	3.5 (0.3)	3.5 (0.4)
CES-D	8.2 (6.4)	10.3 (7.6)	12.9 (9.1)	11.2 (9.4)	7.9 (4.7)	8.3 (6.1)
W-DEQ	65.7 (11.9)	62.5 (13.0)	61.6 (20.8)	67.1 (23.2)	58.0 (12.2)	57.1 (13.4)

^a^At T1/baseline, no statistically significant differences were observed between control and MIL groups

### Childbirth appraisals

#### Childbirth self-efficacy

**Fig. 2:**
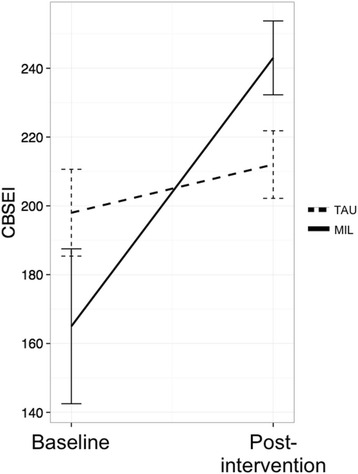
Childbirth Self-Efficacy scores (MIL = Mind in Labor; TAU = Treatment as Usual)

Childbirth self-efficacy (measured with the CBSEI, efficacy subscale) increased by an average of 14.7 points in the TAU group and 78.2 points in the MIL group (see Fig. 2). To assess differential treatment effects, we constructed a linear mixed model with random participant intercepts using R statistical software with the Zelig library [28, 29]. Groups were dummy coded as 0 = TAU and 1 = MIL. There was a significant time*group interaction (*t* = 2.21, *p* = .04), indicating that the MIL group improved significantly more (estimated treatment effect = 64.4 points, 80% CI [26.1, 102.7]). In addition, the effect for time was nonsignificant (*t* = .65, *p* = .52), indicating that the increase in the TAU group was not by itself significantly different from zero. One of the randomized participants was missing a CBSEI score post-intervention. We re-ran these analyses with multiple imputation using the Amelia II library [30] (http://gking.harvard.edu/amelia) with the EM algorithm for estimation. In the imputed dataset the estimated coefficients did not change substantially and the critical time*group interaction remained significant (*t* = 2.18, *p* = .03).

#### Pain catastrophizing

**Fig. 3:**
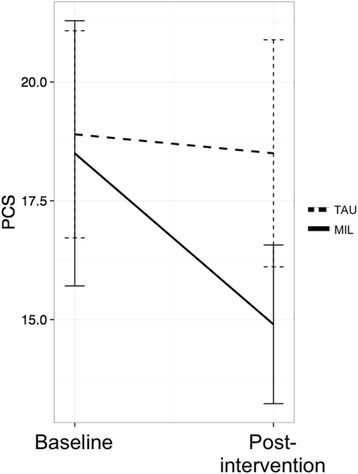
Pain Catastrophizing scores (MIL = Mind in Labor; TAU = Treatment as Usual)

Pain catastrophizing (measured with the PCS) dropped by 3.6 points in the MIL group and was essentially unchanged in the TAU group (see Fig. 3). The time*group interaction was not significant (*t* = -1.06, *p* = .30; estimated treatment effect = -3.26 points, 80% CI [-7.3, 0.8]). When the missing data was imputed, the result did not change (*t* = -.71, *p* = .48).

### Pain medication use in labor

We tested for group differences in use of two types of pain medication during labor. Two participants were excluded from this analysis because they did not experience labor due to scheduled Cesarean. The rate of narcotic use was 30.8% (*n* = 4) in the MIL group and 61.5% (*n* = 8) in the control group. Despite this very large risk ratio (RR = .50), the difference showed only a trend toward statistical significance (Pearson ***χ*** ^**2**^ (df = 1) = 2.48, *p* = .12) (See Table 1). Epidural use was uniformly high and did not differ between groups: 85.7% (*n* = 12) in the MIL group and 84.6% (*n* = 11) in the control group (Pearson ***χ*** ^**2**^ (df = 1) = .01, *p* = .94).

### Restrospective perceived labor pain

For analysis of labor pain, we averaged each participant’s four retrospective VAS scores (early stage, active, pushing, and final). We identified narcotic use, epidural use, pitocin, and birth weight as *a priori* covariates of interest. These covariates were not significantly associated with retrospective perceived pain either singly (all *p* < .36) or in a simultaneous model (multiple R^**2**^ = .19, *p* = .22). Condition was marginally associated with pain, with higher scores for MIL participants (average score of 5.20 on the 1-10 VAS) than for control participants (average score of 3.88; *b* = 1.32, *p* = .07). When condition was entered in a model with the four covariates, its estimated effect dropped slightly (*b* = 1.08, *p* = .14) and the model overall was nonsignificant (multiple R^**2**^ = .29, *p* = .15).

### Body awareness and mindfulness

Body awareness measured by the MAIA scale increased by an average of .10 points (per item) in the TAU group and .56 points in the MIL group. The time*group interaction was significant (*t* = 2.25, *p* = .03; estimated treatment effect = .46 points, 80% CI [.19, .72]). When the missing data was imputed, the treatment effect remained significant (*t* = 2.05, *p* = .04). Mindfulness as measured by the FFMQ scale increased by an average of .09 points (per item) in the TAU group and .08 points in the MIL group. The time*group interaction was not significant (*t* = -.12, *p* = .91; estimated treatment effect = -.01 points, 80% CI [.-16, .13]). When the missing data was imputed, the result did not change (*t* = .01, *p* > .99).

### Perinatal and postpartum depression symptoms

**Fig. 4:**
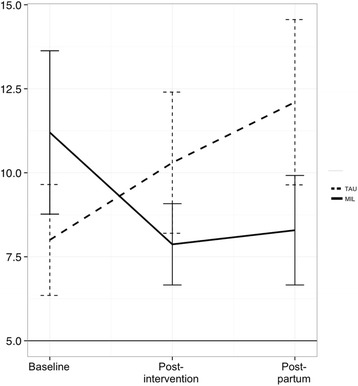
CES-D depression symptom scores (MIL = Mind in Labor; TAU = Treatment as Usual)

Depression symptoms (measured by the CES-D) were analyzed at pre-treatment, post-treatment, and follow-up. Scores dropped by 3.3 points in the MIL condition from pre- to post-treatment, and this improvement was largely maintained at follow-up (see Fig. 4). We constructed a linear mixed model similar to that used for the other questionnaire measures; time was treated as a simple linear factor (coded 0 at pre-treatment, 1 at post-treatment, and 2 at follow-up). The interaction between group and time was significant: *t* = -2.13, *p* = .04. The estimated treatment effect – reflecting the amount by which the two groups diverged at each time point – was -3.34 (80% CI [-5.22, -1.28]). A marginal main effect for time indicated that the increase in depression for the TAU group may have been statistically significant in itself (estimated effect = 2.07 points per time point, *t* = 1.86, *p* = .07). Two CES-D scores were missing; one at post-treatment and one at follow-up. When missing data were imputed, the interaction between group and time remained significant (*t* = -2.12, *p* = .03). [Note. We re-ran these analyses with timepoint as a categorical variable, reflecting the fact that change in depression might not be linear. The results were very similar to those presented here, with significant results for the critical interaction at follow-up and marginally significant results at post-treatment.].

### Birth satisfaction

Birth satisfaction was assessed postnatally using two measures: the W-DEQ and the single-item satisfaction rating. Average W-DEQ scores were 57.1 in the MIL group and 61.6 in the control group, both near the middle of the possible range (0 – 144). The group difference was not significant (*t* = .72, *p* = .48). Scores on the single-item satisfaction rating were very high and likely showed a ceiling effect: the overall mean response was 7.9 on a 1–10 scale, and a plurality of participants in both groups responded with a 9 or 10 (57% of control and 47% of MIL). To deal with the non-normality of the data, we dichotomized responses into <8 vs. ≥8. There was no difference between groups (Pearson ***χ*** ^**2**^ (df = 1) = .51, *p* = .47).

## Discussion

Previous interventions for childbirth pain and fear have focused almost exclusively on: a) pharmacologic pain management strategies that are important to provide as available options under the standard of care but also pose health risks for the mother and fetus [31], and b) childbirth education that is geared toward increasing factual knowledge about the stages and mechanics of labor and birth, potential problems faced in labor and delivery, and medical interventions that are available to address those problems. Childbirth education is the primary mechanism by which pregnant women learn strategies for coping with pain in labor, yet in its standard form it may fail to reduce fear of childbirth [5]. We found that participation in the MIL, a short, time-intensive weekend workshop form of childbirth education that incorporates mindfulness can lead to significant increases in childbirth self-efficacy not found through participation in standard childbirth education programs. These results are consistent with a single-arm trial conducted of a Mindfulness-Based Childbirth Education course in Australia [32] in which participants experienced large improvements in childbirth self-efficacy and fear of childbirth. Approaching labor with a greater sense of self-efficacy may help prevent an array of undesired outcomes in the intrapartum period that were beyond the scope of this small RCT, but that have been documented in prior research (e.g., emergency Cesarean).

While recent advances in labor pain management through medication have been necessary and appropriate, mindfulness interventions offer a promising and potentially appealing, evidence-based, complementary approach that can equip the laboring woman to more accurately appraise her ability to face labor pain, more effectively cope with that pain, and make better reasoned choices about the medical options available to her during birth. We saw no reduction in epidural rate or retrospective reports of perceived pain during labor, but there was a trend toward lower use of opioid analgesia during labor. The systemic opioids commonly used for labor analgesia have side effects that can negatively impact the fetus [33], thus a lower rate of opioid analgesia is highly desirable. The trend we found toward this outcome may be a result of better labor pain coping among the MIL participants, however we were unable to collect real-time data from the labor experience to examine this potential mechanism. In their meta-analysis of 48 published RCTs, Bricker and Lavender [32] report that only 15% included data for opioid safety outcomes. Their review of observational studies highlights the potential for opioids to cause adverse effects in newborns and indicates some data are available that link fetal exposure to opioids during labor with risk of addictive and self-destructive behaviors later in life. MIL offers a childbirth preparation approach to complement pharmacologic strategies for labor pain management that have variable efficacy, can produce potentially harmful side effects, and may be undesirable for some women.

Notably, we found a reduction in depression symptoms that was maintained at postpartum follow-up in the MIL condition whereas the TAU control group experienced a rise in depression symptoms postpartum. Nationally in the U.S., approximately 14% of women experience postpartum depression (PPD) [34], a condition linked with enduring risks to healthy child development extending into adolescence. A robust literature documents the negative impact of PPD on the quality of mother-infant interaction, with studies showing less reciprocal [35] and more intrusive, hostile, disengaged, and withdrawn behavior among mothers with PPD [36, 37]. Many women who experience PPD have also been depressed during pregnancy [34], indicating the benefit of antenatal intervention to prevent PPD as we saw here. Others have developed indicated approaches to managing women’s depression in pregnancy, e.g., with Mindfulness-Based Cognitive Therapy-Perinatal Depression [38, 39] which is promising; our universal prevention approach in the form of childbirth education may reach a broader audience among the general public, reaching women at-risk for PPD who do not self-identify in that way. MIL also includes fathers/partners, which may produce benefit for preventing fathers’ postpartum depression (something not assessed in the current study) but a growing concern [40, 41].

In addition to showing beneficial improvements in childbirth self-efficacy and depression, this study demonstrated feasibility of online/telephone screening and retention of a diverse sample of pregnant women in an RCT of MIL. There was considerable interest in trial participation and even with rigorous RCT inclusion/exclusion criteria that screened out numerous potential participants, we achieved our target sample in the desired time frame. In terms of retention, only three participants left the study before randomization and we had 100% retention from randomization to postpartum follow-up and missing data on only two participants post-course. In informal reports made to the MIL facilitator and the study project manager, participants generally liked the format of a weekend workshop that allowed them to complete their childbirth education all at once versus needing to complete a multi-week course while facing competing demands due to weekday work schedules and other commitments involved in preparing to become new parents.

### Limitations

Our assessment of labor pain was substantially constrained by the limitations of postpartum retrospective report, so it is possible that an ecological momentary assessment protocol during labor may reveal benefits for pain tolerance or sensitivity. The high rate of epidural use further limited the utility of the pain ratings. Since poor data quality regarding length of labor in the medical records prevented us from examining that key potential covariate, we also cannot be sure that the somewhat lower use of opioid analgesia was due to the mindfulness training. However, the use of an active comparison condition in an RCT design suggests the encouraging results on this outcome should be further tested. As with any small pilot study, we were underpowered to detect some of our hypothesized effects and thus we will aim to conduct a fully powered trial in the future.

In examining self-reports of general dispositional mindfulness and mindful body awareness, we saw significant improvements in body awareness but not dispositional mindfulness. The subfacets of mindful body awareness fit much more closely with the logic model for the MIL intervention involving developing mindfulness of bodily sensations. In contrast, it was improbable that we would see an improvement in self-report dispositional mindfulness in a program that did not emphasize development of a formal daily meditation practice. We will aim to more thoroughly investigate these potential mechanisms and other hypothesized secondary outcomes of the PEARLS study in future work.

In this rigorous pilot efficacy study, we employed the intervention developer to deliver the MIL to all intervention condition study participants. While ensuring the high quality of the intervention delivery in the current study, this strategy may pose concerns about future potential for taking MIL to scale. Following expansion and replication of the current findings, we strongly support lines of inquiry that will tackle questions in mindfulness research regarding the attributes, training, and skills needed to effectively teach mindfulness-based programs with potential for public health benefit.

## Conclusions

We have developed a novel approach to fostering adaptive appraisals of childbirth and reducing perinatal depression symptoms using a mindfulness approach. Although a more definitive trial is warranted, the results of this small RCT suggest that by positively impacting labor and birth processes, while also promoting healthy psychological adjustment in the perinatal period, better postpartum outcomes can be expected. We also successfully recruited and retained a more socieconomically and ethnically diverse study sample than is often found in mindfulness trials in the U.S., suggesting potential for generalization of findings and receptivity to the intervention approach as offered in MIL. If the efficacy of MIL is further supported in a full-scale trial, we believe it can be offered as a universal prevention program designed to reduce childbirth fear and improve adjustment across the perinatal and postpartum periods, reduce pain medication use in labor, and prevent postpartum depression. Intervening in this sensitive period of developmental plasticity may produce important long-term health benefits for children and families.

## Abbreviations

CBSEI: Childbirth Self-Efficacy Inventory
CES-D: Center for Epidemiologic Studies Depression Scale
FFMQ: Five Facet Mindfulness Questionnaire
HIPAA: Health insurance portability and accountability act of 1996
MAIA: Multidimensional Assessment of Interoceptive Awareness
MBCP: Mindfulness-Based Childbirth and Parenting
MBSR: Mindfulness-Based Stress Reduction
MIL: Mind in Labor: Working with Pain in Childbirth
PCS: Pain Catastrophizing Scale
PPD: Postpartum depression
RCT: Randomized controlled trial
RR: Risk ratio
SD: Standard deviation
T1: Time 1 (baseline/pre-intervention/pre-test)
T2: Time 2 (post-intervention but pre-birth)
T3: Time 3 (post-birth/postpartum follow-up)
TAU: Treatment as usual
UCSF: University of California, San Francisco
US: United States
VAS: Visual analog scale
W-DEQ: Wijma Delivery Expectancy/Experience Questionnaire
